# Biomechanical analyses of different serve and groundstroke techniques in tennis: A systematic scoping review

**DOI:** 10.1371/journal.pone.0290320

**Published:** 2023-08-17

**Authors:** Johanna Lambrich, Thomas Muehlbauer

**Affiliations:** Division of Movement and Training Sciences/Biomechanics of Sport, University of Duisburg-Essen, Essen, Germany; Università Telematica degli Studi IUL, ITALY

## Abstract

This systematic scoping review aims to summarize findings regarding kinetic, kinematic, and electromyographic analyses of different characteristics (i.e., type/direction and stance style) of the tennis serve and groundstroke. A systematic search of the literature was performed on the databases PubMed, Web of Science, and SportDiscus from their inception date till May 2023. A descriptive analysis of results was conducted. The literature search identified a total of *N* = 899 records, 23 of which met the inclusion criteria and were analysed in this review. A total of 229 participants aged 18 to 62 years participated in the studies. The studies revealed varying results, ranging from significantly lower/higher values to no significant differences between serve/groundstroke characteristics. These inconsistent results may most likely be attributed to discrepancies in the methodological approach such as players’ age (18–62 years), sex (i.e., men only or both sexes), and performance level (i.e., recreational, intermediate, or advanced) as well as the applied measurement devices (i.e., force plate or pressure-detecting insoles; motion capture system, high-speed video recordings, or IMU sensors) and used outcomes (i.e., measured or estimated force etc.). Future research is needed to provide a comprehensive biomechanical analysis of different serve/groundstroke characteristics. Specifically, it is recommended to compare different tennis serve and groundstroke types/directions and stance styles in female and male age-matched players with diverging performance levels (i.e., recreational, intermediate, advanced) using combined (i.e., kinetic, kinematic, and electromyographic) biomechanical analysis.

## Introduction

In tennis, serves and groundstrokes are essential components to be successful [1]. Their execution is influenced by various factors, such as serve/groundstroke type, groundstroke direction, and serve/groundstroke stance style [2, 3]. Within these factors, there are different characteristics that allow variable movement executions. For example, there is a distinction for a) the serve/stroke type between flat, slice, and topspin, for b) the stroke direction between longline and cross-court, and for c) the stance style between foot-up and foot-back serve as well as between open and square groundstroke [4–9]. The aforementioned different characteristics can lead to discrepancies in kinetic and kinematic variables as well as in muscle activation [10]. For instance, significant differences in kinetic and kinematic parameters were reported between the foot-up compared to the foot-back serve stance and between the attacking neutral versus attacking open versus defensive open groundstroke stance [11–13]. Furthermore, there are studies [5, 14] that observed differences in kinematic and electromyographic parameters between the flat versus slice (sidespin) versus kick (topspin) serve and between the flat compared to the topspin forehand groundstroke.

Despite these results from original studies, a systematic overview regarding kinetic, kinematic, and electromyographic analyses of different characteristics (i.e., type/direction and stance style) of the tennis serve and groundstroke is still lacking. Such an overview might be helpful to identify research gaps on the one hand and to derive directions for future studies on the other hand. In addition, replicated findings on significant differences depending on the different characteristic of serve/groundstroke type, groundstroke direction, and serve/groundstroke stance style can be used to derive indications for specific technical and conditioning training programmes. Thus, the aim of the present systematic scoping review was to provide an overview of the current body of evidence regarding biomechanical analyses (i.e., kinetic, kinematic, and electromyographic) of different serve and groundstroke techniques (i.e., stance style, stroke type/direction) in tennis.

## Methods

In line with the PRISMA Extension for Scoping Reviews [15], we conducted a systematic scoping review of the literature. In accordance with Munn et al. [16], this approach was selected aiming to provide an overview of the current evidence in this field of research and to identify current gaps in knowledge. Systematic scoping reviews are conducted in a structured and reproducible manner with the purpose to diminish bias [16] and to cover a relatively broad field of research (i.e., biomechanical analysis of kinetic, kinematic, and electromyographic variables in tennis).

### Search strategy

The systematic literature search was conducted in the electronic databases PubMed, Web of Science, and SPORTDiscus to identify relevant articles for this review. The following Boolean search term was used: tennis AND (plantar pressure OR loading OR ground reaction force OR kinematic OR kinetic OR electromyographic) AND (stroke type OR stroke stance OR stroke direction OR stroke technique OR groundstroke OR forehand OR backhand OR serve OR slice OR topspin OR flat OR twist OR first serve OR second serve OR open stance OR closed stance OR neutral stance OR square stance) NOT table.

**Fig 1:**
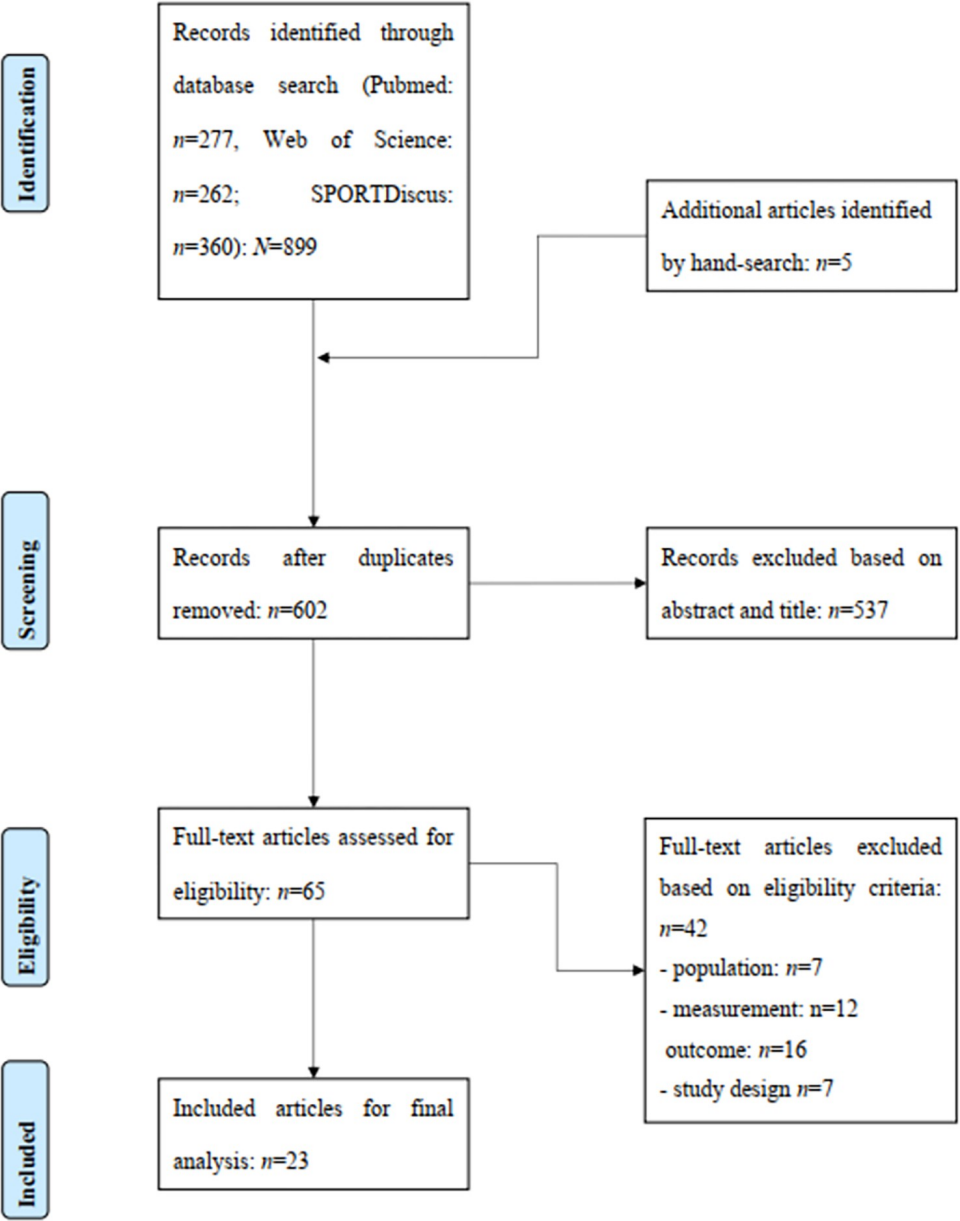
PRISMA flow chart illustrating the different phases of literature search, study selection, and reasons for exclusion of records.

The search covers the period from the inception date to May 2023. The literature search was limited to full texts, English language, and the human species. In addition, reference lists were checked for other relevant publications. After removing all duplicates, the titles and abstracts of all publications were checked by both authors for eligibility according to the inclusion and exclusion criteria (Table 1). The full texts of all potentially eligible studies were assessed according to the criteria. Disagreements were resolved through discussion and consensus. The process of literature search, study selection, and reasons for exclusion of publications are documented in Fig 1 using the PRISMA flow chart.

**Table 1:** Overview of the inclusion and exclusion criteria.

Category	Inclusion criteria	Exclusion criteria
Population	Investigation of healthy adult tennis players	Assessment of beginners, junior/youth, injured tennis players or no tennis players (i.e., table tennis players, overhead athletes)
Measurement	Application of devices to measure kinetic, kinematic, and/or electromyographic parameters for different serve/groundstroke types, groundstroke directions, and/or serve/groundstroke stance styles	Application of performance analysis (e.g., stroke speed) only
Outcome	Analysis at least one kinetic, kinematic, and/or electromyographic outcome	No analysis of influencing factors (i.e., stroke type/direction and stance style) of the tennis serve and groundstroke)
Study design	Use of a cross-sectional or longitudinal study design	Execution of an intervention study without reporting baseline data

### Study selection criteria

The inclusion and exclusion criteria applied are presented in Table 1. To be eligible for inclusion in this review, studies had to meet the following criteria: a) investigation of healthy adult tennis players, b) application of devices to measure kinetic, kinematic, and/or electromyographic parameters for different serve/groundstroke types, groundstroke directions, and/or serve/groundstroke stance styles, c) analysis of at least one kinetic, kinematic, and/or electromyographic outcome, d) use of a cross-sectional or longitudinal study design. Studies were excluded if a) beginners, junior/youth, injured tennis players or no tennis players (i.e., table tennis players, overhead athletes) were tested, b) only a performance analysis (e.g., stroke speed) was performed, c) no influencing factor (i.e., stroke type/direction and stance style) of the tennis serve and groundstroke was investigated, and d) an intervention study without reporting baseline data was conducted.

### Data extraction

Data were extracted relating to author(s) and year of publication; number of tennis players by sex, age, performance level; influencing factor and comparison; measurement and outcomes; results.

## Results

### Study selection

Fig 1 illustrates the process of systematic literature search and study selection. In total, the search identified 899 publications for evaluation. In addition, five studies were added through other sources. After removing the duplicates and checking titles and abstracts, 65 full texts were checked. A total of 42 publications were removed for the following reasons: a) population (*n* = 7), b) measurement (*n* = 12), c) outcome (*n* = 16), and d) study design (*n* = 7).

### Study characteristics

Tables 2 and 3 shows the main characteristics of the included studies for the tennis serve (i.e., type and stance style) and groundstroke (i.e., type, direction, and stance style), respectively. A total of 229 subjects were examined in the included studies. Sixteen studies [4–7, 9, 12–14, 17–24] investigated only male players, while seven papers [8, 11, 21, 25–28] studied mixed players group. Five studies [11, 17–20] did not report the players’ age, and the remaining studies investigated adult players. Ten studies [4, 8, 11, 14, 17–20, 25, 29] analysed the tennis serve, eleven publications [5–7, 9, 12, 13, 21, 23, 24, 27, 28] investigated the forehand groundstroke, and two investigations [22, 26] examined the forehand and backhand groundstroke.

**Table 2:** Chronological overview of the included studies on biomechanical analysis of different tennis serve types and stance styles.

Reference	No. of players; sex; age [years (range or mean ± SD)]; performance level	Factor; comparisons	Measurement: Outcomes	Results
Kinetic measures				
Elliott & Wood [11]	9; F (3), M (6); N/A; A-Grade	*Stance style*: Foot-up vs. foot-back stance	*Force plate*: Vertical and horizontal force	Stance style: • sign. higher vertical force in the foot-up vs. foot-back stance • no sign. differences between stance styles for the horizontal force
Bahamonde & Knudson [17]	9; M; N/A; advanced	*Stance style*: Foot-up vs. foot-back stance *Serve type*: Flat vs. slice (sidespin) serve	*Force plate*: Vertical and horizontal force	Stance style: sign. higher peak vertical force and horizontal braking force in the foot-up vs. foot-back stance • no sign. differences between stance styles in peak forward propulsive force Serve type: • no sign. differences between serve types in ground reaction forces
Girard et al. [8]	10; F (3); M (7); 23.8 ± 6 years; competitive (ITN 3)	*Stance style*: Foot-up vs. foot-back stance *Serve type*: Flat (first) vs. twist (second) serve	*Insole plantar pressure system*: Maximum and mean vertical force; peak and mean pressure	Stance style: • sign. higher loading on the lateral forefoot but lower loads on the medial heel of the front foot in the foot-up vs. foot-back stance • sign. higher loading on the lateral mid-foot but lower on the medial forefoot of the back foot in the foot-up vs. foot-back stance Serve type: • sign. higher peak and mean pressures as well as maximum and mean forces under the lateral forefoot of the front foot in the flat vs. twist serve • no sign. differences between serve types for the backfoot in pressure and force values
Kinematic measures				
Elliott & Wood [11]	9; F (3), M (6); N/A; A-Grade	*Stance style*: Foot-up vs. foot-back stance	*High-speed video recordings*: Racket velocity; Angular displacement and velocity for the knee, hip, shoulder, elbow, wrist	Stance style: • sign. higher range of shoulder movement and average angular velocity of the shoulder for the foot-up vs. foot-back stance • no sign. difference between stance styles in racket, wrist, elbow, shoulder, hip, and knee angles at impact • no sign. difference between stance styles in serve velocity
Reid et al. [18]	12; M; N/A; high-performance	*Serve type*: Flat vs. kick (topspin) serve	Motion analysis system: Racket velocity; Angular displacement and velocity for the shoulder, trunk, hip, and knee	*Serve type*: • sign. higher peak horizontal, vertical, and absolute pre-impact racket velocities in the flat vs. kick serve • sign. lower lateral racket velocities at impact in the flat vs. kick serve • sign. higher shoulder alignment left lateral flexion in the flat vs. kick serve • sign. lower shoulder alignment right rotation and forward flexion in the flat vs. kick serve • sign. lower peak front knee joint extension angular velocity and maximum rear hip vertical velocity in the flat vs. kick serve • no sign. differences between serve types for maximum external rotation of the racket shoulder, upper arm plane of elevation angle, peak shoulder joint internal rotation angular velocity, upper arm–thorax elevation angle, and left lateral flexion shoulder–pelvis alignment separation angle
Reid et al. [19]	12; M; N/A; high-performance	*Stance style*: Foot-up vs. foot-back stance	Motion analysis system: Angular displacement and velocity of the shoulder	Stance style: • no sign. differences between stance styles in shoulder joint kinematics (i.e., peak shoulder joint internal rotation angular velocity, upper arm–thorax elevation angle, lateral flexion separation angle, range of shoulder alignment lateral flexion, range of shoulder alignment rotation)
Chow et al. [14]	19; M; 25.3 ± 4.1 years / 23.4 ± 6.5 years; intermediate (*n* = 8; NTRP = 4.5–5.0) / advanced (*n* = 11; NTRP = 5.5)	*Serve type*: Flat vs. slice (sidespin) vs. kick (topspin) serve	Motion analysis system: Angular displacement for trunk extension, left lateral flexion, left/right twisting	*Serve type*: • no sign. differences between serve types in trunk motions
Sheets et al. [4]	7; M; 18–22 years; NCAA Division I collegiate tennis players	*Serve type*: Flat vs. slice (sidespin) vs. kick (topspin) serve	Motion analysis system: Racket velocity; velocity for the wrist, elbow, shoulder, and lower back	*Serve type*: • no sign. differences between serve types in racket velocity at ball impact • sign. larger lateral component and lower forward component of the racket velocity vector for the kick vs. flat and slice serve • sign. lower vertical component of the racket velocity vector for the flat vs. slice and kick serve • no sign. differences between serve types for the peak velocity or velocity at impact of the elbow, shoulder, or back • sign. faster wrist velocity for the flat vs. slice and kick serve
Martin et al. [25]	15; F (4), M (11); 25 ± 6.1 years; expert	*Stance style*: Foot-up vs. foot-back stance	Motion analysis system: Ball velocity	*Stance style*: • sign. higher post-impact ball velocity for the foot-up vs. foot-back stance
Abrams et al. [20]	7; M; N/A; NCAA Division I collegiate tennis players	*Serve type*: Flat vs. slice (sidespin) vs. kick (topspin) serve	Motion analysis system: Angular displacement of the wrist, elbow, shoulder, and lower back	Serve type: • sign. higher shoulder internal rotation velocity for the flat vs. slice and kick serve • no sign. differences between serve types in shoulder external rotation angle, back extension, and internal/external rotation extension angle
Electromyographical measures				
Chow et al. [29]	19; M; 25.3 ± 4.1 years / 23.4 ± 6.5 years; intermediate (*n* = 8; NTRP = 4.5–5.0) / advanced (*n* = 11; NTRP = 5.5)	*Serve type*: Flat vs. slice (sidespin) vs. kick (topspin) serve	Electromyography: Activation of the rectus abdominis left/right, external oblique left/right, internal oblique left/right, erector spinae left/right	*Serve type*: • no sign. differences between the serve types in trunk muscle activation

Note. F: female

ITN: International Tennis Number

M: Male

N/A: not available

NCAA: National Collegiate Athletic Association

NTRP: United States Tennis Association National Tennis Rating Program (range from 1.0 [beginner] to 7.0 [world class professional]).

**Table 3:** Chronological overview of the included studies on biomechanical analysis of different tennis groundstroke types, directions, and stance styles.

Reference	No. of players; sex; age [years (range or mean ± SD)]; performance level	Factor; comparisons	Measurement: Outcomes	Results
*Kinetic measures*				
Martin et al. [12]	8; M; 26.3 ± 11.0 years; advanced (ITN 4–5)	*Stance style*: Attacking neutral (ANS) vs. attacking open (AOS) vs. defensive open stance (DOS) forehand	*Force plate*: Anterior, lateral, and vertical force	Stance style: • sign. higher lateral force in the DOS vs. AOS and ANS • sign. higher lateral force in the AOS vs. ANS • sign. higher vertical force in the DOS vs. ANS
Martin et al. [13]	8; M; 26.3 ± 11.0 years; advanced (ITN 4–5)	*Stance style*: Attacking neutral (ANS) vs. attacking open (AOS) vs. defensive open stance (DOS) forehand	*Force plate*: Anterior, lateral, and vertical force	Stance style: • sign. higher lateral force in the DOS vs. AOS and ANS • sign. higher lateral force in the AOS vs. ANS • sign. higher vertical force in the DOS vs. ANS
Lambrich & Muehlbauer [26]	39; F (22), M (17); 17–32 years; recreational (*n* = 13), intermediate (*n* = 13), advanced (*n* = 13)	*Stroke direction*: Longline vs. cross forehand and backhand strokes	*Insole plantar pressure system*: Maximum force [N/kg], force-time integral [Ns/kg]	Stroke direction: • no sign. differences between stroke directions in force values during forehand stroke • sign. higher maximum force in the rearfoot during the cross vs. longline backhand stroke
Kinematic measures				
Elliott & Marsh [27]	7; F (1), M (6); 19–26 years; competitive (national)	*Stroke type*: Topspin vs. backspin forehand down-the-line	*High-speed video recordings*: Angular displacement and velocity of the racket, knee, hip, shoulder, elbow, and wrist	Stroke type: • sign. smaller shoulder but larger racket angle for the topspin vs. backspin at the completion of the backswing • no sign. differences between stroke types in left and right knee, right hip, elbow, and wrist angle at the completion of the backswing sign. higher racket velocity for the topspin vs. backspin at pre- and post-impact • sign. higher wrist velocity for the topspin vs. backspin during the forward swing • sign. larger left and right knee and right hip angles for the topspin vs. backspin at impact • sign. larger left and right knee, right hip, shoulder, elbow, and wrist velocities for the topspin vs. backspin at impact
Elliott et al. [5]	12; M; 22 years; high-performance	*Stroke type*: Flat vs. topspin forehand drive vs. topspin lob	*High-speed video recordings*: Angular displacement and velocity for the racket, shoulder, upper arm, forearm, hand	Stroke type: • sign. higher velocity of the racket head, shoulder, and upper arm (i.e., horizontal flexion/abduction; internal rotation) at impact in horizontal (forward) direction for the flat forehand drive vs. topspin forehand drive and topspin lob • sign. lower velocity of the racket head and the shoulder at impact in the horizontal (sideward) direction for the flat forehand drive vs. topspin forehand drive and topspin lob • no sign. differences between stroke types in joint angles at impact and completion of the backswing
Knudson & Bahamonde [21]	11; F (2), M (9); 21–62 years; intermediate (*n* = 8), professional (*n* = 7)	*Stance style*: Open vs. square stance forehand drive	*High-speed video recordings*: Racket velocity; Angular velocity for the trunk	*Stance style*: • no sign. differences between stance styles in racket velocity, vertical path of the racket, and trunk angular velocity at impact
Landlinger et al. [6]	13; M; 16–25 years; high-performance (*n* = 7), elite (*n* = 6)	*Stroke direction*: Longline vs. cross-court forehand stroke	Motion analysis system: Racket velocity; Angular displacement and velocity for the trunk, pelvis, hip, shoulder, elbow, and wrist	*Stroke direction*: • sign. larger racket angle and hip alignment for the longline vs. cross-court stroke • sign. lower separation angle, horizontal racket velocity, and pelvis rotation velocity for the longline vs. cross-court stroke • sign. later occurrence of peak elbow velocity for the longline vs. cross-court stroke
Landlinger et al. [7]	13; M; 16–25 years; high-performance (*n* = 7), elite (*n* = 6)	*Stroke direction*: Longline vs. cross-court forehand stroke	Motion analysis system: Racket velocity; Angular displacement and velocity for the trunk, pelvis, hip, shoulder, elbow, and wrist	*Stroke direction*: • sign. lower separation angles for the longline vs. cross-court stroke at end of backswing • sign. lower racket velocity and further front alignment for the hip, shoulder, and racket for the longline vs. cross-court stroke at impact • no sign. differences between stroke directions in shoulder velocity at impact • sign. larger hip and shoulder alignment angles for the longline vs. cross-court stroke in the follow through phase
Cabral [22]	5; M; 31.2 ± 7.32 years; competitive (ATP within the top 100)	*Stance style*: Open vs. closed stance forehand/backhand stroke	*Motion analysis system*: Angular displacement for the shoulder	Stance style: • sign. lower post-impact ball speed and shoulder rotation angle during forehand and backhand stroke in the open vs. closed stance
Kawamoto et al. [9]	13; M; 25.0 ± 2.5 years; advanced (ITN 2–4)	*Stance style*: Open vs. square stance forehand topspin stroke	*Motion analysis system*: Racket velocity; Angular velocities for the torso, pelvis, shoulder, upper arm, elbow, and wrist	*Stance style*: • no sign. differences between stance styles in racket velocity • sign. shorter duration from pelvis forward rotation to ball impact in the open vs. square stance style • sign. lower peak velocity of torso’s centre of mass and of the shoulder joint centre in the hitting direction for the open vs. square stance style • no sign. difference between stance styles in the peak upper arm and elbow/wrist joint angular velocity
Genevois et al. [23]	14; M; 29.3 ± 7.0 years; advanced (ITN 1–3)	*Stroke type*: Flat vs. topspin forehand stroke	*Motion analysis system*: Racket velocity; Angular displacement and velocities for the thorax, shoulder, elbow, and wrist	Stroke type: • sign. lower post-impact ball velocity and horizontal racket velocity but higher vertical racket velocity for the topspin vs. flat stroke • sign. higher flexion and adduction of the humerothoracic joint, pronation of the elbow joint, and extension of the wrist joint at the beginning of the forehand for the topspin vs. flat stroke • sign. higher pronation of the elbow joint at the completion of the backswing for the topspin vs. flat stroke • sign. higher external rotation of the humerothoracic joint at impact for the topspin vs. flat stroke • sign. higher abduction and internal rotation of the humerothoracic joint and pronation of the elbow joint at the completion of the follow-through phase for the topspin vs. flat stroke
Martin et al. [12]	8; M; 26.3 ± 11.0 years; advanced (ITN ≥4)	*Stance style*: Attacking neutral (ANS) vs. attacking open (AOS) vs. defensive open stance (DOS) forehand	*Motion analysis system*: Angular displacement of the hip	*Stance style*: • sign. higher minimal and maximal hip flexion angles in the DOS vs. AOS and ANS • sign. higher minimal and maximal hip abduction angles in the DOS vs. ANS and in ANS vs. AOS • sign. higher maximal hip external rotation in the DOS vs. ANS
Martin et al. [13]	8; M; 26.3 ± 11.0 years; advanced (ITN 4–5)	*Stance style*: Attacking neutral (ANS) vs. attacking open (AOS) vs. defensive open stance (DOS) forehand	*Motion analysis system*: Angular displacement of the knee	*Stance style*: • sign. higher maximal knee flexion and minimal knee abduction in DOS vs. AOS and ANS • sign. higher maximal knee abduction in DOS vs. ANS and in AOS vs. ANS
Pedro et al. [24]	6; M; 21.0 ± 4.2 years; national (*n* = 3), professional (*n* = 3)	*Stroke direction*: Inside-out vs. cross-court forehand stroke	*IMU sensors*: Angular displacements and velocities for the shoulder, elbow, wrist	*Stroke direction*: • sign. higher shoulder alignment for inside-out vs. cross-court stroke at end of the back swing • sign. lower shoulder alignment for inside-out vs. cross-court stroke at impact • no sign. differences between stroke directions in elbow and wrist angles, irrespective of stroke phase • no sign. differences between stroke directions in shoulder, elbow, and wrist velocity
Electromyographical measures				
Knudson & Blackwell [28]	14; F (6), M (8); 20.4 ± 2.6 years; collegiate	*Stance style*: Open stance vs. square stance forehand	*Electromyography*: Activation of the rectus abdominis, external oblique, erector spinae	Stance style: • no sign. difference between stance styles in muscle activation

Note. ANS: attacking neutral stance

AOS: attacking open stance

ATP: Association of Tennis Professionals

DOS: defensive open stance

F: female

IMU: inertial measurement unit

ITN: International tennis number

M: male.

### Biomechanical analysis of different tennis serve types and stance styles

#### Kinetic analysis of different serve types

Two studies [8, 17] compared different types of tennis serve using kinetic parameters. Bahamonde and Knudson [17] examined nine male advanced tennis players and reported no significant differences in ground reaction forces assessed via two force plates between the flat versus slice (sidespin) serve. Further, Girard et al. [8] investigated ten female and male competitive tennis players and used plantar pressure-detecting insoles. They found that the mean and peak pressures as well as the mean and maximal forces were significantly higher under the lateral forefoot of the front foot in flat (first) compared to the twist (second) tennis serve. However, no significant differences in pressure and force values were detected between the flat versus twist serve of the backfoot.

#### Kinematic analysis of different serve types

Four studies [4, 14, 18, 20] analysed kinematic parameters to compare different serve types. Reid et al. [18] examined twelve right-handed male high-performance tennis players using a Vicon motion analysis system and reported significantly higher peak horizontal, vertical, and absolute pre-impact racket velocities during the flat compared to the kick (topspin) serve, while lateral racket velocities at impact were significantly lower for the flat compared to the kick serve. In terms of body kinematics, the authors reported that shoulder alignment left lateral flexion was significantly higher, but shoulder alignment right rotation and forward flexion were significantly lower in the flat compared to the kick serve. Further, peak front knee joint extension angular velocity and maximum rear hip vertical velocity were significantly lower in the flat compared to the kick serve. However, no significant differences between the flat and slice serve were detected for all other kinematic measures. Additionally, Chow et al. [14] used video cameras to capture racket and whole-body movements and studied eight intermediate and eleven advanced male tennis players. They found no significant differences between the flat, slice (sidespin), and kick (topspin) serve for the analysed four trunk motions (i.e., extension, left lateral flexion, left and right twisting). Moreover, Sheets and colleagues [4] investigated seven male NCAA Division I collegiate tennis players using a motion capture system. In terms of racket velocity, the authors reported no significant differences at ball impact between the serve types (i.e., flat vs. slice [sidespin] vs. kick [topspin] serve). However, they found significant differences in the direction of the racket velocity vector between the serve types, i.e., largest lateral and smallest forward components for the kick serve and smallest vertical component for the flat serve. Concerning body kinematics, there were no significant differences between serve types for the peak speed or speed at impact of the elbow, shoulder, or back. Yet, there was only a significant difference for the wrist, that was fastest during the flat serve. Lastly, Abrams et al. [20] studied seven male NCAA Division I collegiate tennis players that performed the flat, slice (sidespin), and kick (topspin) serve. The authors detected significantly higher shoulder internal rotation velocity for the flat compared to the slice and kick serve. For all other kinematic variables (i.e., back extension and internal/external rotation extension angle, shoulder external rotation angle), no significant differences between serves were obtained in the angle values.

#### Electromyographical analysis of different serve types

Only one study [14] compared different types of tennis serve using electromyographic parameters. In addition to the above-mentioned kinematic analysis of trunk motion, Chow et al. [14] also examined trunk muscle activity (i.e., rectus abdominis, external and internal oblique, erector spinae) and observed no significant differences between the flat, slice (sidespin), and kick (topspin) serve.

#### Kinetic analysis of different serve stance styles

Three studies [8, 11, 17] compared different serve stance styles using kinetic parameters. Elliot and Wood [11] used a force plate and examined nine A-grade tennis players (3 females, 6 males). They reported significantly higher maximum force levels for the vertical direction of the ground reaction force for the foot-up compared to the foot-back stance but no significant differences between stance styles in the horizontal forward-backward direction of the ground reaction force. In addition to the above-mentioned kinetic analysis of different serve types, Bahamonde and Knudson [17] also compared different serve stances and observed significantly higher peak vertical ground reaction force and horizontal braking force for the foot-up compared to the foot-back stance but no significant differences between stance styles in peak forward propulsive force. Further, Girard et al. [8] investigated not only different serve types but also discrepancies in two serve stance styles. The authors reported that the loading was significantly higher on the lateral forefoot but lower on the medial heel of the front foot with foot-up compared to foot-back stance. Further, loading of the back foot was significantly higher under the lateral mid-foot but lower under the medial forefoot when using a foot-up versus foot-back stance.

#### Kinematic analysis of different serve stance styles

Three studies [11, 19, 25] analysed kinematic parameters to compare different stance styles during the tennis serve. In addition to the above-mentioned kinetic analysis, Elliot and Wood [11] also used high-speed video recordings and observed a significantly higher range of shoulder movement and average angular velocity of the shoulder for the foot-up compared to the foot-back stance technique but no significant differences in serve velocity as well as racket, wrist, elbow, shoulder, hip, and knee angles at impact. Furthermore, Reid et al. [19] examined twelve high-performance male tennis players and reported no significant differences in shoulder joint kinematics (i.e., peak shoulder joint internal rotation angular velocity, upper arm–thorax elevation angle, lateral flexion separation angle, range of shoulder alignment lateral flexion, range of shoulder alignment rotation) between the foot-up compared to the foot-back stance. In addition, Martin et al. [25] investigated 15 expert female and male tennis players and detected significantly higher post-impact ball velocity for the foot-up than the foot-back stance.

#### Electromyographic analysis of different serve stance styles

So far no study exists that used parameters of muscle activation to analyse different serve stance styles, which highlights the need to conduct such investigations in the future.

### Biomechanical analysis of different groundstroke types, directions, and stances styles

#### Kinetic analysis of different groundstroke types

To the best of our knowledge, there is no study available that used kinetic parameters to analyse different groundstroke types, indicating a crucial research gap for future studies.

#### Kinematic analysis of different groundstroke types

Three studies [5, 23, 27] analysed kinematic parameters to compare different groundstroke types. Elliott and Marsh [27] examined seven (1 female, 6 male) competitive tennis players using three-dimensional high-speed video cameras and reported significantly higher racket velocity (at pre- and post-impact) for the topspin compared to the backspin forehand stroke. At the completion of the backswing, a significantly smaller angle for the shoulder during the topspin versus backspin stroke but no differences for the left and right knee, right hip, elbow, and wrist angles were found. During the forward swing, the authors detected significantly higher velocities for the wrist but not for the elbow and shoulder joint for the topspin when compared with the backspin stroke. Lastly, they reported significantly larger angular displacements (left and right knee, right hip) and velocities (left and right knee, right hip, shoulder, elbow, and wrist) for the topspin versus backspin at impact. In another study, Elliott et al. [5] again used high-speed cinematographic technique to analyse racket and upper-limb movements in twelve high-performance male tennis players. At impact, they detected significantly higher velocities for the racket head, shoulder, and upper arm (i.e., horizontal flexion/abduction; internal rotation) in the horizontal (forward) direction (forward) for the flat forehand drive versus topspin forehand drive and topspin lob. Conversely, significantly lower velocities of the racket head and shoulder in the horizontal (sideward) direction were found for flat forehand drive than the topspin forehand drive and the topspin lob. Yet, no significant differences were observed between stroke types in joint angles at impact and completion of the backswing. Lastly, Genevois et al. [23] studied 14 male competitive tennis players that performed flat and topspin forehand groundstrokes. The authors detected significantly lower post-impact ball velocity and horizontal racket velocity but higher vertical racket velocity for the topspin compared to the flat stroke. At the beginning of the forehand drive, they reported significantly higher flexion and adduction of the humerothoracic joint, pronation of the elbow joint as well as higher extension of the wrist joint for the topspin than for the flat stroke. Concerning the completion of the backswing, significantly higher pronation of the elbow joint for the topspin when compared to flat stroke was found. At impact, the authors observed significantly higher external rotation of the humerothoracic joint for the topspin versus the flat stroke. Regarding the completion of the follow-through phase, significantly higher abduction and internal rotation of the humerothoracic joint and pronation of the elbow joint was shown for the topspin when compared to the flat forehand stroke.

#### Electromyographic analysis of different groundstroke types

There is currently no study available that compared different types of tennis groundstrokes using electromyographic parameters.

#### Kinetic analysis of different groundstroke directions

There is only one study [26] that compared different groundstroke directions using kinetic parameters. Precisely, Lambrich and Muehlbauer [26] investigated 39 female and male tennis players of different performance levels using plantar pressure-detecting insoles. They found significantly higher maximum force values in the rearfoot during cross-court compared to longline backhand strokes. However, no significant differences in force values were detected between stroke directions during forehand stroke.

#### Kinematic analysis of different groundstroke directions

Three studies [6, 7, 24] performed kinematic analyses of different groundstroke directions in tennis. Landlinger et al. [6, 7] examined 13 male tennis players of different performance levels (elite and high-performance) using a motion capture system. In the first study [6], they reported a) significantly larger racket angle and hip alignment, b) significantly lower separation angle, horizontal racket velocity, and pelvis rotation velocity, and c) significantly later occurrence of peak elbow velocity for the longline versus cross-court stroke direction. In the second study [7], the authors detected significantly lower separation angles for the longline compared to the cross-court stroke direction at end of backswing. At impact, they found a significantly lower racket velocity and a further front alignment for the hip, shoulder, and racket during the longline when compared with the cross-court stroke direction. However, their analyses yielded no significant differences in shoulder velocity between stroke directions at impact. At the end of forward racquet movement (i.e., follow through phase), they observed significantly larger hip and shoulder alignment angles for the longline than the cross-court stroke direction. Further, Pedro et al. [24] investigated six male national and professional tennis players using inertial sensors. The authors reported significantly higher shoulder alignment for the inside-out compared to the cross-court stroke direction at end of the back swing but significantly lower shoulder alignment for the inside-out than the cross-court direction at impact. However, no significant differences were detected in elbow and wrist angles as well as in shoulder, elbow, and wrist velocity between stroke directions.

#### Electromyographic analysis of different groundstroke directions

Up to date, there is no study available that used parameters of muscle activation to analyse different groundstroke directions, which indicates a further research gap for future studies.

#### Kinetic analysis of different groundstroke stances

Two studies [12, 13] analysed kinetic parameters to compare different groundstroke stances. Martin et al. [12, 13] examined eight male advanced players using a motion capture system and reported significantly higher lateral ground reaction forces in the defensive open compared to the attacking open and attacking neutral stance as well as in the attacking open versus attacking neutral stance. In addition, vertical ground reaction forces were significantly higher in the defensive open than in the attacking neutral stance.

#### Kinematic analysis of different groundstroke stances

Five studies [9, 12, 13, 21, 22] analysed kinematic parameters to compare different groundstroke stances. Knudson and Bahamonde [21] measured racket and trunk kinematics in eleven intermediate and professional tennis players of both sexes using high-speed video recordings and reported no significant differences in racket velocity, vertical path of the racket, and trunk angular velocity at impact between the open compared to the square stance. Further, Cabral [22] investigated five male professional tennis players that performed open and closed stance forehand and backhand groundstrokes. For both strokes, the analyses showed that post-impact ball velocity and angle of shoulder rotation were significantly lower in the open than the closed stance. Moreover, Kawamoto et al. [9] studied 13 advanced male tennis players and observed significantly shorter duration from pelvis forward rotation to ball impact in the open compared to the square stance. Additionally, the authors reported significantly lower peak velocity of the torso’s centre of mass and the shoulder joint centre in the hitting direction during open versus square stance. However, no significant differences were detected in racket velocity and in the peak upper arm as well as in elbow and wrist joint angular velocity between open and square stance. Lastly and in addition to the above-mentioned kinetic analysis of different groundstroke stances, Martin et al. [12, 13] also examined hip and knee kinematics. Regarding the hip, they found a) significantly higher minimal and maximal flexion angles in the defensive open compared to the attacking open and attacking neutral stance, b) significantly higher minimal and maximal abduction angles in the defensive open than the attacking neutral stance and in the attacking neutral versus attacking open stance, and c) significantly higher maximal external rotation in the defensive open compared with the attacking neutral stance. Concerning the knee, the authors detected a) significantly higher maximal flexion and minimal abduction in the defensive open compared to the attacking open and attacking neutral stance and b) significantly higher maximal abduction in the defensive open than the attacking neutral stance and in the attacking open versus attacking neutral stance.

#### Electromyographic analysis of different groundstroke stances

Only one study [28] compared different types of groundstroke stances using electromyographic parameters. Specifically, Knudson and Blackwell [28] examined trunk muscle activation (i.e., rectus abdominis, external oblique, erector spinae) in 14 female and male collegiate tennis players and observed no significant differences in electromyography of the trunk muscles between the open compared to the square stance forehand.

## Discussion

The present systematic scoping review provides an overview over the current body of evidence regarding biomechanical analyses (i.e., kinetic, kinematic, and electromyographic) of different serve and groundstroke techniques in tennis. Overall, the analyses for both serve and groundstroke techniques revealed mostly significant differences in kinetic, kinematic, and electromyographical variables as a function of the factors serve/groundstroke type, groundstroke direction, and serve/groundstroke stance style. However, the evidence remains limited as it was shown how widely the studies’ applied methodology differs in terms of sample size (i.e., 5–39 players), participants characteristics (i.e., age range, performance level, and sex distribution), applied measurement devices (i.e., motion capture system, high-speed video recordings, IMU sensors etc.), and analysed outcome measures (i.e., kinetics, kinematics, muscle activation). In addition, research gaps were revealed, as no studies have been conducted on some factors so far. Specifically, there is a lack of kinetic and electromyographic analyses of different groundstroke types as well as of electromyographic analyses of different groundstroke directions and serve stance styles.

### Biomechanical analysis of different serve techniques

In a total of six studies [4, 8, 14, 17, 18, 20], either two (i.e., flat vs. slice or flat vs. twist) or three (flat vs. slice [sidespin] vs. kick [topspin]) serve types were compared with each other. Of these, two studies [8, 17] conducted kinetic analyses only while another three studies [4, 18, 20] performed kinematic analyses only. One study [14] combined kinematic and electromyographic analyses. The kinetic analyses revealed no significant differences in force values between the flat and slice serve [17]. The study by Girard et al. [8] showed significant differences between serves but does not allow a comparison with the previously mentioned study, as other serve types (i.e., flat versus twist) were examined. The kinematic analyses showed an inconsistent picture despite similar outcome measures. For example, Reid et al. [18] detected significantly higher (i.e., horizontal and vertical) as well as lower (i.e., lateral) racket velocities for the flat compared to the kick serve, but Sheets et al. [4] found no significant differences between serve types. In addition, Sheets and colleagues reported no significant differences between serve types for shoulder velocity, but Abrams et al. [20] observed a significantly higher shoulder internal rotation velocity for the flat compared to the slice and kick serve. One explanation could relate to discrepancies between the methodological approaches (e.g., players’ characteristics). Further, the combined kinematic and electromyographic analyses yielded no significant differences between serve types in trunk motion and trunk muscle activation [14]. Therefore, future studies comparing different types of tennis serves should include age-matched players with diverging performance levels and apply a combination of kinetic, kinematic, and electromyographic analyses.

A total of five studies [8, 11, 17, 19, 25] compared the foot-up with the foot-back serve stance style. Of these, two studies performed only a kinetic [8, 17] or kinematic [19, 25] analysis and one study [11] combined kinetic and kinematic analyses. The kinetic analyses yielded relatively homogeneous findings with significantly higher vertical forces for the foot-up compared to the foot-back serve stance style and no significant differences between the two styles for the forward forces. Therefore, it can be concluded that the foot-up stance style supports the generation of vertical forces that positively effects post-impact ball velocity [25]. Contrary, the kinematic analyses showed heterogeneous results ranging from significant differences (i.e., higher post-impact ball velocity and higher movement range and angular velocity of the shoulder) in favour of the foot-up stance style [11, 25] to no significant differences between stance styles [11, 19]. One reason could be that the players’ performance level differed (i.e., A-grade, high-performance, or expert) between studies which, despite the same serve stance style, can result in different outcomes [30]. Therefore, future investigations should compare serve stance style (i.e., foot-up versus foot-back) in players with diverging performance levels (i.e., recreational, intermediate, and advanced) within one study. In this regard, a combination of kinetic kinematic, and electromyographic analyses should be applied, as no study exists for the analysis of muscle activation.

### Biomechanical analysis of different groundstroke techniques

Three studies [5, 23, 27] compared either two (i.e., topspin vs. backspin forehand or flat vs. topspin forehand) or three (i.e., flat forehand vs. topspin forehand vs. topspin lob) groundstroke types. All of these studies [5, 23, 27] conducted only kinematic analyses but no study used kinetic analyses. Genevois et al. [23] examined different groundstroke types (i.e., flat vs. topspin forehand) and reported significantly lower horizontal but higher vertical racket velocity for the topspin compared to the flat forehand. Based on the observed differences between topspin and flat forehand, it can be deduced that specific instructional practices as well as technical training and conditioning programs are necessary for optimal performance in the respective groundstroke type.

Regarding groundstroke direction, one study [26] conducted only kinetic, three studies [6, 7, 24] applied only kinematic and no study performed electromyographic or combined analyses. Three studies [6, 7, 26] compared longline with cross-court direction and one study [24] inside-out with cross-court direction. The kinetic analysis revealed significantly higher maximum force values in the rearfoot during the cross-court compared to the longline backhand stroke and no significant differences in force values between stroke directions during the forehand stroke. This suggests that different stroke directions have only a limited impact on force generation. Therefore, direction-specific exercises seem to play a minor role in technical training routines. A direct comparison of the kinematic analyses can only be made in two cases [6, 7], as the authors compared the same groundstroke directions (i.e., longline vs. cross-court forehand). In both studies, the authors reported significantly lower racket velocity and separation angle as well as larger hip alignment for the longline compared to the cross-court direction. These findings suggest that, with regard to racket and body kinematics, direction-specific exercises are important to achieve optimal stroke performance for both techniques. Unfortunately, it is not possible to make further statements about electromyographic and combined analyses, as such investigations are still lacking.

Concerning groundstroke stance styles, three studies [9, 21, 22] applied only kinematic, one study [28] conducted only electromyographic, two studies [12, 13] performed combined (i.e., kinetic and kinematic), and no study used only kinetic analyses. Four studies [9, 21, 22, 28] compared two (i.e., open vs. square stance or open vs. closed stance) and two studies [12, 13] contrasted three (i.e., attacking neutral vs. attacking open vs. defensive open stance) stance styles. The kinetic analysis of Martin et al. [12, 13] showed significantly higher lateral and vertical ground reaction forces in the defensive open compared to the attacking neutral stance. Additionally, the lateral ground reaction forces were significantly higher in the attacking open when compared with the attacking neutral stance. The additionally performed kinematic analysis yielded significantly higher joint angles for the hip (i.e., flexion, abduction, and external rotation) and knee (i.e., flexion and abduction) in the defensive open compared to the attacking open and attacking neutral stance. The results of these two studies suggest that the greater muscular loading in terms of strength and flexibility during the defensive open stance can lead to a greater risk of injury. Therefore, the authors recommended to avoid this stance style. Alternatively, appropriate strength and flexibility conditioning programs could be performed to reduce the risk of hip and knee injury. A further direct comparison of the kinematic analyses can be made for the open versus square stance style. Both studies [9, 21] reported no significant differences between the two stances in racket velocity as well as in trunk [21] and upper-body [9] kinematics. Thus, it can be concluded that the stance style has no influence on the stroke technique. This interpretation is supported by the only electromyographic analysis [28] that also reported no significant differences between open versus square stance. Thus, the application of stance-specific stroke drills does not seem to have a significant role in stroke performance.

### Limitations

The present systematic scoping review has a few limitations. The methodology of the included studies varied in terms of players’ performance level (i.e., recreational, intermediate, or advanced), setting (i.e., laboratory conditions or field-based testing), measurement (e.g., force plate or pressure-detecting insoles; motion capture system, high-speed video recordings, or IMU sensors), and outcomes (i.e., kinetic [force, pressure], kinematic, or electromyographic values). Further, the included studies were conducted with healthy tennis players in the age range of 18–62 years only, thus no statements can be made about younger or older players. Moreover, in 16 out of 23 included studies only male players were tested and thus some statements are limited to this gender.

## Conclusion

Different serve and groundstroke techniques are considered to have a substantial impact of stroke performance. Despite a large number of original studies, a summarising overview with regard to biomechanical analyses is still missing. This systematic scoping review for the first time summarised kinetic, kinematic, and electromyographic analyses of different characteristics (i.e., stroke type/direction and stance style) of the tennis serve and groundstroke. On the one hand, our analyses yielded varying results that included significant or no significant differences between the aforementioned characteristics. Most likely, methodological differences are responsible for this, as a) forces were measured (i.e., by means of a force plate) or estimated (i.e., by means of pressure-detecting insoles), b) youth or adult tennis players were studied, and c) recreational, intermediate, or advanced players were tested. On the other hand, research gaps were identified that revealed a lack of electromyographic analyses for the stance style during tennis serve and for stroke direction and type during groundstroke. Further, no kinetic analysis was found for different groundstroke types. Based on these findings, it is recommended that future studies should investigate the characteristics of different serve and groundstroke types/directions and stance styles of age-matched players with diverging performance levels (i.e., recreational, intermediate, advanced) using combined (i.e., kinetic, kinematic, and electromyographic) analyses.

## Supporting information

S1 Checklist PRISMA-checklist–transparent reporting of systematic reviews and meta-analyses. (DOC)
